# Supplementary material for: Heparin-based hydrogel scaffolding alters the transcriptomic profile and increases the chemoresistance of MDA-MB-231 triple-negative breast cancer cells
Source: Biomater Sci. 2020 Feb 13;8(10):2786–96. doi: 10.1039/c9bm01481k (PMC7497406; doi:10.1039/c9bm01481k)
Supplement: Supplementary file 2 [file BM-008-C9BM01481K-s002.zip › Supplementary File 4/HP-B hydrogel + EGF (s)vControl/Pathways/my_analysis.Gsea.1545201170987/HALLMARK_E2F_TARGETS.html]

Details for gene set HALLMARK\_E2F\_TARGETS[GSEA]

|  || Dataset | expr.class.cls#HYDEGF\_versus\_CONTROL.class.cls#HYDEGF\_versus\_CONTROL\_repos |
| Phenotype | class.cls#HYDEGF\_versus\_CONTROL\_repos |
| Upregulated in class | HYDEGF |
| GeneSet | HALLMARK\_E2F\_TARGETS |
| Enrichment Score (ES) | 0.28925648 |
| Normalized Enrichment Score (NES) | 1.3151469 |
| Nominal p-value | 0.040453073 |
| FDR q-value | 0.12852675 |
| FWER p-Value | 0.816 |
Table: GSEA Results Summary

  

Fig 1: Enrichment plot: HALLMARK\_E2F\_TARGETS      
 Profile of the Running ES Score & Positions of GeneSet Members on the Rank Ordered List

  

| PROBE | DESCRIPTION (from dataset) | GENE SYMBOL | GENE\_TITLE | RANK IN GENE LIST | RANK METRIC SCORE | RUNNING ES | CORE ENRICHMENT || 1 | DDX39A | na |  |  | 115 | 2.860 | 0.0143 | Yes |
| 2 | CENPM | na |  |  | 184 | 2.633 | 0.0295 | Yes |
| 3 | AURKB | na |  |  | 223 | 2.534 | 0.0456 | Yes |
| 4 | DNMT1 | na |  |  | 248 | 2.469 | 0.0619 | Yes |
| 5 | TACC3 | na |  |  | 271 | 2.421 | 0.0780 | Yes |
| 6 | CIT | na |  |  | 337 | 2.327 | 0.0912 | Yes |
| 7 | ESPL1 | na |  |  | 387 | 2.248 | 0.1046 | Yes |
| 8 | TIMELESS | na |  |  | 613 | 1.995 | 0.1070 | Yes |
| 9 | KIF18B | na |  |  | 632 | 1.976 | 0.1201 | Yes |
| 10 | MCM2 | na |  |  | 778 | 1.847 | 0.1256 | Yes |
| 11 | CDKN2C | na |  |  | 781 | 1.845 | 0.1387 | Yes |
| 12 | NOP56 | na |  |  | 837 | 1.790 | 0.1485 | Yes |
| 13 | TCF19 | na |  |  | 899 | 1.752 | 0.1578 | Yes |
| 14 | WEE1 | na |  |  | 1066 | 1.644 | 0.1608 | Yes |
| 15 | MYBL2 | na |  |  | 1167 | 1.589 | 0.1668 | Yes |
| 16 | HMGB2 | na |  |  | 1230 | 1.550 | 0.1746 | Yes |
| 17 | EZH2 | na |  |  | 1241 | 1.545 | 0.1851 | Yes |
| 18 | MCM7 | na |  |  | 1322 | 1.505 | 0.1916 | Yes |
| 19 | POLA2 | na |  |  | 1347 | 1.495 | 0.2010 | Yes |
| 20 | POLD1 | na |  |  | 1350 | 1.493 | 0.2116 | Yes |
| 21 | POLE | na |  |  | 1398 | 1.468 | 0.2195 | Yes |
| 22 | PNN | na |  |  | 1542 | 1.412 | 0.2221 | Yes |
| 23 | KIF22 | na |  |  | 1674 | 1.366 | 0.2249 | Yes |
| 24 | CDC25A | na |  |  | 1707 | 1.357 | 0.2329 | Yes |
| 25 | CDCA8 | na |  |  | 1723 | 1.351 | 0.2418 | Yes |
| 26 | SPAG5 | na |  |  | 1844 | 1.312 | 0.2448 | Yes |
| 27 | GINS3 | na |  |  | 1913 | 1.288 | 0.2504 | Yes |
| 28 | TK1 | na |  |  | 2001 | 1.265 | 0.2548 | Yes |
| 29 | ILF3 | na |  |  | 2042 | 1.253 | 0.2617 | Yes |
| 30 | RAD50 | na |  |  | 2063 | 1.248 | 0.2695 | Yes |
| 31 | PLK4 | na |  |  | 2424 | 1.146 | 0.2587 | Yes |
| 32 | LIG1 | na |  |  | 2535 | 1.119 | 0.2609 | Yes |
| 33 | SMC1A | na |  |  | 2558 | 1.113 | 0.2677 | Yes |
| 34 | MCM5 | na |  |  | 2694 | 1.082 | 0.2683 | Yes |
| 35 | CDCA3 | na |  |  | 2993 | 1.005 | 0.2598 | Yes |
| 36 | CCNE1 | na |  |  | 3012 | 1.001 | 0.2660 | Yes |
| 37 | TBRG4 | na |  |  | 3040 | 0.995 | 0.2716 | Yes |
| 38 | MCM4 | na |  |  | 3125 | 0.976 | 0.2742 | Yes |
| 39 | MCM3 | na |  |  | 3325 | 0.938 | 0.2704 | Yes |
| 40 | WDR90 | na |  |  | 3383 | 0.926 | 0.2740 | Yes |
| 41 | SUV39H1 | na |  |  | 3419 | 0.918 | 0.2787 | Yes |
| 42 | H2AFX | na |  |  | 3433 | 0.916 | 0.2845 | Yes |
| 43 | STAG1 | na |  |  | 3553 | 0.892 | 0.2846 | Yes |
| 44 | DIAPH3 | na |  |  | 3708 | 0.863 | 0.2827 | Yes |
| 45 | ATAD2 | na |  |  | 3782 | 0.850 | 0.2849 | Yes |
| 46 | UBE2S | na |  |  | 3814 | 0.843 | 0.2893 | Yes |
| 47 | PRPS1 | na |  |  | 4047 | 0.804 | 0.2828 | No |
| 48 | E2F8 | na |  |  | 4157 | 0.790 | 0.2827 | No |
| 49 | TRA2B | na |  |  | 4229 | 0.777 | 0.2845 | No |
| 50 | MYC | na |  |  | 4314 | 0.764 | 0.2855 | No |
| 51 | SMC4 | na |  |  | 4414 | 0.747 | 0.2856 | No |
| 52 | DONSON | na |  |  | 4539 | 0.729 | 0.2843 | No |
| 53 | CDC20 | na |  |  | 4567 | 0.724 | 0.2880 | No |
| 54 | UNG | na |  |  | 4743 | 0.698 | 0.2838 | No |
| 55 | HELLS | na |  |  | 4989 | 0.653 | 0.2756 | No |
| 56 | SPC24 | na |  |  | 5156 | 0.629 | 0.2713 | No |
| 57 | NCAPD2 | na |  |  | 5324 | 0.600 | 0.2668 | No |
| 58 | CDC25B | na |  |  | 5390 | 0.592 | 0.2676 | No |
| 59 | GINS1 | na |  |  | 5489 | 0.577 | 0.2665 | No |
| 60 | RFC2 | na |  |  | 5525 | 0.573 | 0.2688 | No |
| 61 | NUP153 | na |  |  | 5529 | 0.572 | 0.2727 | No |
| 62 | BARD1 | na |  |  | 5616 | 0.559 | 0.2722 | No |
| 63 | NOLC1 | na |  |  | 5664 | 0.551 | 0.2736 | No |
| 64 | PRIM2 | na |  |  | 5670 | 0.551 | 0.2773 | No |
| 65 | KIF2C | na |  |  | 5742 | 0.538 | 0.2774 | No |
| 66 | DSCC1 | na |  |  | 5784 | 0.534 | 0.2790 | No |
| 67 | PPM1D | na |  |  | 6219 | 0.474 | 0.2596 | No |
| 68 | ASF1B | na |  |  | 6363 | 0.456 | 0.2553 | No |
| 69 | AK2 | na |  |  | 6374 | 0.455 | 0.2580 | No |
| 70 | BRCA2 | na |  |  | 6406 | 0.449 | 0.2596 | No |
| 71 | MXD3 | na |  |  | 6534 | 0.433 | 0.2560 | No |
| 72 | BRCA1 | na |  |  | 6597 | 0.424 | 0.2557 | No |
| 73 | DCTPP1 | na |  |  | 6695 | 0.412 | 0.2536 | No |
| 74 | PRKDC | na |  |  | 6828 | 0.394 | 0.2494 | No |
| 75 | RANBP1 | na |  |  | 6835 | 0.393 | 0.2519 | No |
| 76 | PPP1R8 | na |  |  | 7042 | 0.367 | 0.2437 | No |
| 77 | ING3 | na |  |  | 7073 | 0.362 | 0.2447 | No |
| 78 | PSMC3IP | na |  |  | 7101 | 0.358 | 0.2458 | No |
| 79 | GINS4 | na |  |  | 7127 | 0.356 | 0.2470 | No |
| 80 | POLE4 | na |  |  | 7207 | 0.342 | 0.2453 | No |
| 81 | SMC6 | na |  |  | 7210 | 0.342 | 0.2477 | No |
| 82 | ZW10 | na |  |  | 7329 | 0.325 | 0.2438 | No |
| 83 | CHEK1 | na |  |  | 7331 | 0.325 | 0.2460 | No |
| 84 | PLK1 | na |  |  | 7470 | 0.309 | 0.2410 | No |
| 85 | KIF4A | na |  |  | 7539 | 0.300 | 0.2395 | No |
| 86 | CENPE | na |  |  | 7608 | 0.290 | 0.2380 | No |
| 87 | SRSF1 | na |  |  | 7695 | 0.280 | 0.2355 | No |
| 88 | TFRC | na |  |  | 7904 | 0.255 | 0.2264 | No |
| 89 | POLD2 | na |  |  | 7915 | 0.254 | 0.2276 | No |
| 90 | PAN2 | na |  |  | 7961 | 0.248 | 0.2270 | No |
| 91 | ASF1A | na |  |  | 8185 | 0.227 | 0.2169 | No |
| 92 | TUBG1 | na |  |  | 8505 | 0.186 | 0.2015 | No |
| 93 | PSIP1 | na |  |  | 8547 | 0.181 | 0.2006 | No |
| 94 | RACGAP1 | na |  |  | 8576 | 0.177 | 0.2004 | No |
| 95 | ORC2 | na |  |  | 8584 | 0.176 | 0.2013 | No |
| 96 | RFC3 | na |  |  | 8598 | 0.174 | 0.2018 | No |
| 97 | RPA1 | na |  |  | 8622 | 0.168 | 0.2018 | No |
| 98 | MTHFD2 | na |  |  | 8784 | 0.152 | 0.1945 | No |
| 99 | SNRPB | na |  |  | 8812 | 0.145 | 0.1941 | No |
| 100 | MLH1 | na |  |  | 8880 | 0.138 | 0.1915 | No |
| 101 | CHEK2 | na |  |  | 9065 | 0.120 | 0.1827 | No |
| 102 | BIRC5 | na |  |  | 9147 | 0.110 | 0.1792 | No |
| 103 | LMNB1 | na |  |  | 9148 | 0.110 | 0.1800 | No |
| 104 | TRIP13 | na |  |  | 9171 | 0.106 | 0.1796 | No |
| 105 | SSRP1 | na |  |  | 9282 | 0.091 | 0.1745 | No |
| 106 | EED | na |  |  | 9293 | 0.089 | 0.1746 | No |
| 107 | NUP107 | na |  |  | 9297 | 0.089 | 0.1750 | No |
| 108 | NUP205 | na |  |  | 9529 | 0.071 | 0.1634 | No |
| 109 | MMS22L | na |  |  | 9713 | 0.048 | 0.1541 | No |
| 110 | PDS5B | na |  |  | 9810 | 0.034 | 0.1493 | No |
| 111 | RRM2 | na |  |  | 9839 | 0.030 | 0.1480 | No |
| 112 | POLD3 | na |  |  | 9852 | 0.029 | 0.1476 | No |
| 113 | TIPIN | na |  |  | 10059 | 0.011 | 0.1369 | No |
| 114 | NASP | na |  |  | 10061 | 0.011 | 0.1369 | No |
| 115 | BRMS1L | na |  |  | 10170 | -0.005 | 0.1312 | No |
| 116 | USP1 | na |  |  | 10213 | -0.009 | 0.1291 | No |
| 117 | ORC6 | na |  |  | 10250 | -0.016 | 0.1273 | No |
| 118 | MCM6 | na |  |  | 10326 | -0.026 | 0.1235 | No |
| 119 | MELK | na |  |  | 10765 | -0.075 | 0.1010 | No |
| 120 | SHMT1 | na |  |  | 10813 | -0.079 | 0.0991 | No |
| 121 | CDKN3 | na |  |  | 10895 | -0.093 | 0.0955 | No |
| 122 | CCNB2 | na |  |  | 11220 | -0.126 | 0.0794 | No |
| 123 | AURKA | na |  |  | 11282 | -0.134 | 0.0771 | No |
| 124 | LUC7L3 | na |  |  | 11305 | -0.137 | 0.0769 | No |
| 125 | PMS2 | na |  |  | 11428 | -0.150 | 0.0716 | No |
| 126 | RAD51AP1 | na |  |  | 11430 | -0.150 | 0.0726 | No |
| 127 | NME1 | na |  |  | 12003 | -0.217 | 0.0441 | No |
| 128 | DEK | na |  |  | 12009 | -0.218 | 0.0454 | No |
| 129 | EXOSC8 | na |  |  | 12137 | -0.234 | 0.0403 | No |
| 130 | XRCC6 | na |  |  | 12225 | -0.249 | 0.0375 | No |
| 131 | CDK4 | na |  |  | 12236 | -0.251 | 0.0388 | No |
| 132 | LBR | na |  |  | 12297 | -0.261 | 0.0375 | No |
| 133 | PHF5A | na |  |  | 12400 | -0.272 | 0.0341 | No |
| 134 | NBN | na |  |  | 12404 | -0.272 | 0.0359 | No |
| 135 | H2AFZ | na |  |  | 12572 | -0.292 | 0.0291 | No |
| 136 | TOP2A | na |  |  | 12732 | -0.319 | 0.0231 | No |
| 137 | XPO1 | na |  |  | 13014 | -0.343 | 0.0107 | No |
| 138 | BUB1B | na |  |  | 13024 | -0.345 | 0.0127 | No |
| 139 | DLGAP5 | na |  |  | 13044 | -0.348 | 0.0142 | No |
| 140 | SLBP | na |  |  | 13063 | -0.352 | 0.0157 | No |
| 141 | MSH2 | na |  |  | 13118 | -0.356 | 0.0154 | No |
| 142 | RAD1 | na |  |  | 13176 | -0.365 | 0.0150 | No |
| 143 | NUDT21 | na |  |  | 13214 | -0.373 | 0.0158 | No |
| 144 | RNASEH2A | na |  |  | 13230 | -0.375 | 0.0176 | No |
| 145 | GSPT1 | na |  |  | 13252 | -0.378 | 0.0192 | No |
| 146 | CCP110 | na |  |  | 13280 | -0.382 | 0.0205 | No |
| 147 | HMMR | na |  |  | 13307 | -0.387 | 0.0219 | No |
| 148 | PA2G4 | na |  |  | 13412 | -0.405 | 0.0193 | No |
| 149 | CTCF | na |  |  | 13593 | -0.423 | 0.0129 | No |
| 150 | DCLRE1B | na |  |  | 13825 | -0.462 | 0.0040 | No |
| 151 | DEPDC1 | na |  |  | 13857 | -0.468 | 0.0057 | No |
| 152 | TMPO | na |  |  | 13866 | -0.469 | 0.0086 | No |
| 153 | UBR7 | na |  |  | 13919 | -0.474 | 0.0093 | No |
| 154 | PAICS | na |  |  | 14009 | -0.490 | 0.0081 | No |
| 155 | RAD51C | na |  |  | 14225 | -0.509 | 0.0004 | No |
| 156 | UBE2T | na |  |  | 14265 | -0.515 | 0.0020 | No |
| 157 | RAN | na |  |  | 14371 | -0.532 | 0.0003 | No |
| 158 | LYAR | na |  |  | 14656 | -0.582 | -0.0105 | No |
| 159 | CKS1B | na |  |  | 14664 | -0.584 | -0.0067 | No |
| 160 | SRSF2 | na |  |  | 14778 | -0.601 | -0.0084 | No |
| 161 | CSE1L | na |  |  | 14801 | -0.604 | -0.0052 | No |
| 162 | CDKN1A | na |  |  | 14882 | -0.616 | -0.0051 | No |
| 163 | PCNA | na |  |  | 14963 | -0.629 | -0.0048 | No |
| 164 | POP7 | na |  |  | 14987 | -0.631 | -0.0015 | No |
| 165 | MAD2L1 | na |  |  | 15013 | -0.635 | 0.0017 | No |
| 166 | RFC1 | na |  |  | 15216 | -0.672 | -0.0041 | No |
| 167 | RAD21 | na |  |  | 15296 | -0.686 | -0.0034 | No |
| 168 | NAA38 | na |  |  | 15366 | -0.699 | -0.0020 | No |
| 169 | SMC3 | na |  |  | 15589 | -0.740 | -0.0084 | No |
| 170 | CDKN1B | na |  |  | 15677 | -0.758 | -0.0076 | No |
| 171 | ANP32E | na |  |  | 15680 | -0.758 | -0.0023 | No |
| 172 | PRDX4 | na |  |  | 15690 | -0.760 | 0.0026 | No |
| 173 | RBBP7 | na |  |  | 15942 | -0.808 | -0.0048 | No |
| 174 | CDK1 | na |  |  | 15956 | -0.811 | 0.0003 | No |
| 175 | SYNCRIP | na |  |  | 16017 | -0.824 | 0.0030 | No |
| 176 | IPO7 | na |  |  | 16235 | -0.881 | -0.0022 | No |
| 177 | RPA2 | na |  |  | 16249 | -0.886 | 0.0035 | No |
| 178 | EIF2S1 | na |  |  | 16348 | -0.909 | 0.0048 | No |
| 179 | NAP1L1 | na |  |  | 16373 | -0.918 | 0.0101 | No |
| 180 | CBX5 | na |  |  | 16382 | -0.919 | 0.0162 | No |
| 181 | KPNA2 | na |  |  | 16498 | -0.947 | 0.0169 | No |
| 182 | PTTG1 | na |  |  | 16987 | -1.085 | -0.0011 | No |
| 183 | RPA3 | na |  |  | 17084 | -1.110 | 0.0018 | No |
| 184 | HUS1 | na |  |  | 17253 | -1.162 | 0.0013 | No |
| 185 | TP53 | na |  |  | 17262 | -1.165 | 0.0091 | No |
| 186 | DUT | na |  |  | 17427 | -1.226 | 0.0092 | No |
| 187 | STMN1 | na |  |  | 17644 | -1.311 | 0.0072 | No |
| 188 | DCK | na |  |  | 18375 | -1.733 | -0.0188 | No |
| 189 | HNRNPD | na |  |  | 18418 | -1.769 | -0.0084 | No |
| 190 | SPC25 | na |  |  | 18500 | -1.837 | 0.0004 | No |
| 191 | CKS2 | na |  |  | 18969 | -2.501 | -0.0064 | No |
| 192 | HMGB3 | na |  |  | 19000 | -2.585 | 0.0104 | No |
Table: GSEA details [plain text format]

  

Fig 2: HALLMARK\_E2F\_TARGETS      
 Blue-Pink O' Gram in the Space of the Analyzed GeneSet

  

Fig 3: HALLMARK\_E2F\_TARGETS: Random ES distribution      
 Gene set null distribution of ES for **HALLMARK\_E2F\_TARGETS**
